# The effects of Ai Chi for balance in individuals with chronic stroke: a randomized controlled trial

**DOI:** 10.1038/s41598-020-58098-0

**Published:** 2020-01-27

**Authors:** Pei-Hsin Ku, Szu-Fu Chen, Yea-Ru Yang, Ta-Chang Lai, Ray-Yau Wang

**Affiliations:** 1 Department of Physical Therapy and Assistive Technology, National Yang-Ming University, Taipei, Taiwan, ROC; 2 Department of Physical Medicine and Rehabilitation, Cheng Hsin General Hospital, Taipei, Taiwan, ROC; 3 Department of Neurology, Cheng Hsin General Hospital, Taipei, Taiwan, ROC

**Keywords:** Randomized controlled trials, Stroke

## Abstract

This study investigated the effectiveness of Ai Chi compared to conventional water-based exercise on balance performance in individuals with chronic stroke. A total of 20 individuals with chronic stroke were randomly allocated to receive either Ai Chi or conventional water-based exercise for 60 min/time, 3 times/week, and a total of 6 weeks. Balance performance assessed by limit of stability (LOS) test and Berg balance scale (BBS). Fugl-Meyer assessment (FMA) and gait performance were documented for lower extremity movement control and walking ability, respectively. Excursion and movement velocity in LOS test was significantly increased in anteroposterior axis after receiving Ai Chi (p = 0.005 for excursion, p = 0.013 for velocity) but not conventional water-based exercise. In particular, the improvement of endpoint excursion in the Ai Chi group has significant inter-group difference (p = 0.001). Both groups showed significant improvement in BBS and FMA yet the Ai Chi group demonstrated significantly better results than control group (p = 0.025). Ai Chi is feasible for balance training in stroke, and is able to improve weight shifting in anteroposterior axis, functional balance, and lower extremity control as compared to conventional water-based exercise.

## Introduction

Stroke is a cerebral vascular disease caused by the interruption of the blood supply to the brain, cutting off the supply of oxygen and nutrients^1^. Damage to the brain tissue leads to sensory, motor, cognitive, and emotional deficits. With impaired motor and sensory functions, stroke patients suffer from deficits in balance control which plays crucial role in ambulatory function and thus as an important clinical indicator^2–5^. Balance is defined as the ability to maintain center of mass (COM) within the stability limits, the boundaries of the base of support (BOS)^6^. Balance control can be quantified by limit of stability (LOS) test, expressed by movement velocity, displacement excursion, and directional control^7,8^. Individuals with stroke usually show decline in the abovementioned balance performance^9–12^. Bohannon^13^ noted the correlation between static standing ability and independent mobility in stroke patients (r = 0.62). Lee *et al*.^14^ found that walking velocity is associated with maximal displacement excursion in LOS test (r = 0.68, p < 0.01) and Berg balance scale (r = 0.66, p < 0.01) in patients with stroke. In addition, the balance-related fall risks should also be addressed in people with chronic stroke^15,16^. Therefore, it is crucial to improve balance control in order to improve the balance-related activities for individuals with stroke.

Several elements, such as strengthening, postural control, weight shifting, and agility exercise, are necessary to be incorporated during balance training^17^. It has also been noted that increased somatosensory inputs and visual deprivation might exert positive effects on top of balance training, as well as enriched environment^4,5,18,19^. Water-based exercise, by utilizing the properties of water, including buoyancy, viscosity, turbulence, and hydrostatic pressure, has been suggested to improve balance control^20,21^. Two reviews summarized that the water-based exercise for neurological disorder covers a wide variety, including resistance training, movement facilitation, motor control training, balance training, coordination training and other specific techniques^21,22^. They indicated that stroke patients improved significantly more in weight shifting ability, dynamic balance, and functional mobility as compared with the land-based intervention^21,22^.

Ai Chi, first developed by Jun Konno in 1990s^23^, is one kind of water-based exercise emphasizing characteristics of balance training^24^. It resembles Tai Chi on land, complemented by Zen shiatzu and Watsu concepts^25^. Ai Chi is composed of 16 katas (movements), including breathing, upper extremity movements, lower extremity movements, trunk control, and coordinated movements^23^. With the properties and advantages of water, less weight bearing is required and larger displacement can be achieved. Currently, some studies have mentioned the benefits of Ai Chi for neurological involved patients^21,22^. Bayraktar *et al*. showed positive effects of 8 weeks of Ai Chi training on muscle strength, muscle endurance, functional mobility, and fatigue severity in patients with multiple sclerosis^26^. Noh *et al*. found that the balance performance and knee flexors strength improved more in the Ai Chi combining Halliwick therapy group than the conventional physiotherapy group in patients with stroke^27^. Pérez-de la Cruz *et al*. also showed the feasibility of Ai Chi on balance and functional capacity for people with Parkinson’s disease^28^.

Taking together, water-based exercise is beneficial for balance performance in patients with stroke. Ai Chi is a specific water-based exercise which emphasizes the characteristics of balance control. However, whether Ai Chi can exert better effect on balance performance than conventional water-based exercise in people with stroke is not known. The aim of this study was to compare the effects of Ai Chi training with conventional water-based exercise on balance performance in people with stroke. We hypothesized that Ai Chi can result in superior effects on balance control than conventional water-based exercise people with stroke.

## Results

### Flow of participants through the study

**Figure 1:**
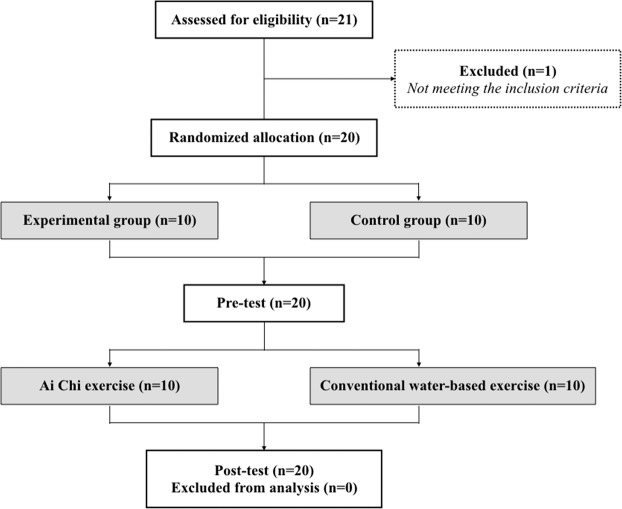
Study flow chart.

Recruitment occurred between April 2016 and September 2017 according to the Institutional Review Board (IRB) approval period. There were 20 participants (14 males and 6 females with mean age of 54.6 years old) provided informed consent and were randomly assigned to the experimental or the control group (n = 10 for each group) (Fig. 1). There were no significant differences in baseline demographic information between the experimental and the control groups (Tables 1 and 2).

**Table 1:** Baseline demographic and clinical characteristics of the participants.

	Ai Chi group (n = 10)	Control group (n = 10)	P value
Age, y	55 (7.3)	52.5 (6.3)	0.970
Gender, male/female	7/3	7/3	1.000
Height, cm	165.0(15.8)	165.5 (8.0)	0.632
Body weight, kg	69.0 (24.5)	66.5 (11.8)	0.705
Side of hemiparesis, right/left	3/7	4/6	0.639
Type of stroke, ischemic/hemorrhagic/mixed	7/2/1	6/2/2	0.815
Time poststroke, month	9.5 (11.0)	22.0 (19.8)	0.255
Weight distribution in standing			
Affected side (% body weight)	50.0 (9.0)	45.0 (11.0)	0.739
Non-affected side (% body weight)	50.0 (9.0)	56.0 (11.0)	0.739
Walking aids used, yes/no	2/8	3/7	0.606
Rehabilitation ongoing, yes/no	3/7	7/3	0.074
MMSE	29.0 (2.5)	29.0 (1.8)	0.796

Values are median and interquartile range for continuous variables and frequency for categorical variables

Abbreviation: MMSE, mini-mental state examination.

**Table 2:** Baseline data for balance, gait, and motor control.

	Ai Chi group (n = 10)	Control group (n = 10)	P value
***LOS test***			
**Endpoint excursion (%)**			
Anteroposterior	72.6 ± 13.0	80.2 ± 21.4	0.545
Lateral	114.9 ± 33.9	108.6 ± 22.9	0.496
**Maximal excursion (%)**			
Anteroposterior	102.0 ± 25.0	104.8 ± 32.8	0.520
Lateral	146.4 ± 36.3	138.6 ± 30.0	0.325
**Directional control (%)**			
Anteroposterior	62.5 ± 14.0	67.6 ± 9.2	0.677
Lateral	77.4 ± 12.6	82.2 ± 10.5	0.241
**Movement velocity (deg/s)**			
Anteroposterior	2.2 ± 1.1	2.2 ± 1.2	1.000
Lateral	3.8 ± 2.5	3.3 ± 1.6	0.762
***Gait performance***			
Speed (cm/s)	61.8 ± 14.6	60.0 ± 27.5	0.650
Cadence (steps/min)	82.7 ± 14.9	88.1 ± 28.3	0.450
Stride length (cm)	82.1 ± 22.9	77.3 ± 20.8	0.597
Stride time (sec)	1.5 ± 0.3	1.7 ± 1.1	0.623
Stride length variability (%)	6.8 ± 2.7	6.7 ± 3.1	0.848
Stride time variability (%)	7.7 ± 4.0	7.6 ± 4.9	0.593
Spatial asymmetry ratio	1.1 ± 0.2	1.1 ± 0.1	0.344
Temporal asymmetry ratio	1.3 ± 0.3	1.3 ± 0.4	0.940
***BBS score***	45.9 ± 5.2	48.7 ± 5.2	0.796
***FMA score***	22.0 ± 3.9	21.7 ± 5.7	0.853

Values are mean ± standard deviation

Abbreviation: LOS, limit of stability; BBS, Berg balance scale; FMA, Fugl-Meyer assessment.

**Figure 2:**
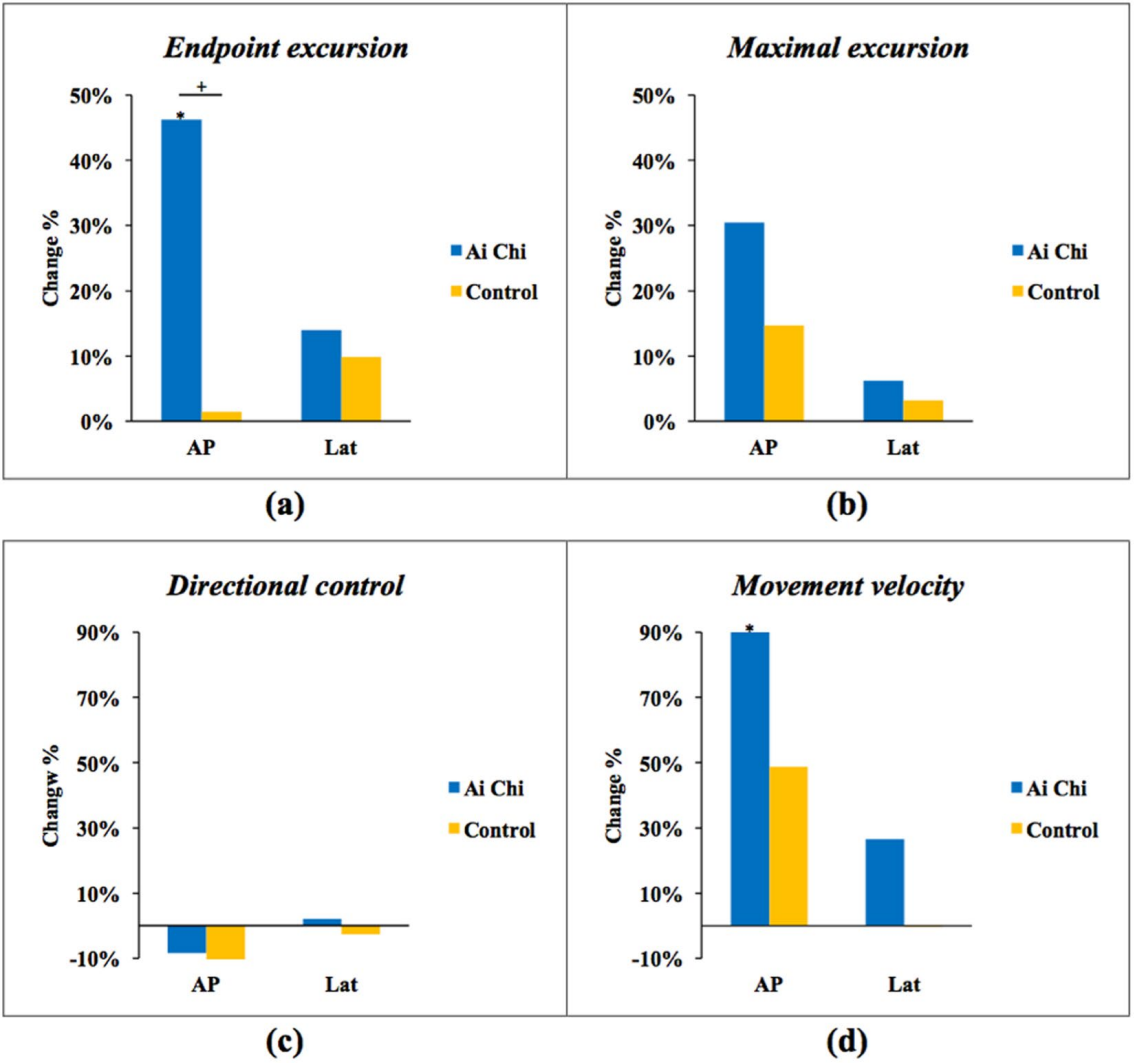
Change of LOS in anteroposterior (AP) and lateral (Lat) direction in Ai Chi and Control group: (**a**) endpoint excursion (**b**) maximal excursion (**c**) directional control (**d**) movement velocity.

The results of dynamic balance indicated by LOS are shown in Table 3 and Fig. 2. Within-group comparisons revealed significant improvements in endpoint excursion (EPE, p = 0.005), maximal excursion (MXE, p = 0.007), and movement velocity (MVL, p = 0.013) in anteroposterior direction after Ai Chi training (Fig. 2a,b,d). Ai Chi training also significantly improved the EPE in anteroposterior direction as compared to the control training (p = 0.001). There were no significant changes in LOS test after control training.

**Table 3:** Dynamic balance performance: limit of stability (LOS) test.

	Ai Chi group (n = 10)			Control group (n = 10)		
	Pre	Post	Change value^a^	Pre	Post	Change value^a^
**Endpoint excursion (%)**						
Anteroposterior	72.6 ± 13.0	105.0 ± 22.6**	46.2 ± 29.8%^++^	80.2 ± 21.4	80.5 ± 21.2	1.5 ± 19.1%
Lateral	114.9 ± 33.9	125.6 ± 28.7	14.0 ± 23.9%	108.6 ± 22.9	118.5 ± 24.3	9.9 ± 14.2%
**Maximal excursion (%)**						
Anteroposterior	102.0 ± 25.0	132.1 ± 35.2**	30.5 ± 22.0%	104.8 ± 32.8	116.4 ± 31.0	14.7 ± 20.8%
Lateral	146.4 ± 36.3	153.6 ± 34.9	6.2 ± 9.3%	138.6 ± 30.0	142.6 ± 30.9	3.2 ± 12.2%
**Directional control (%)**						
Anteroposterior	62.5 ± 14.0	53.3 ± 16.8	−8.4 ± 43.1%	67.6 ± 9.2	57.3 ± 17.3	−15.1 ± 23.4%
Lateral	77.4 ± 12.6	76.7 ± 8.8	2.1 ± 23.2%	82.2 ± 10.5	80.35 ± 13.2	−2.6 ± 9.1%
**Movement velocity (deg/s)**						
Anteroposterior	2.2 ± 1.1	3.4 ± 1.1*	91.2 ± 104.6%	2.2 ± 1.2	2.5 ± 1.2	48.7 ± 78.3%
Lateral	3.8 ± 2.5	4.7 ± 2.0	26.5 ± 67.2%	3.3 ± 1.6	2.9 ± 1.2	−0.3 ± 41.6%

Values are mean ± standard deviation.

^a^Change values were calculated by subtracting the pre-training data from the post-training data divided by pre-training data.

*, **p < 0.05, p < 0.01 for intra-group comparison.

^++^p < 0.01 for inter-group comparison.

Regarding the functional balance performance, both groups improved significantly in Berg Balance Scale (BBS) scores after training (p = 0.005, p = 0.043, for Ai Chi and control group respectively) with no significant group difference (Table 4). However, 7 out of 10 subjects improved at least 4 points (minimal detectable change, MDC^29^) after Ai Chi training, while 2 out of 10 improved beyond the MDC after control training (p = 0.025 for group comparison).

**Table 4:** Functional balance performance indicated by Berg balance scale (BBS).

	Ai Chi (n = 10)		Control (n = 10)	
	Pre	Post	Pre	Post
BBS score	45.9 ± 5.2	51.1 ± 4.1*	48.7 ± 5.2	51.2 ± 3.9*
Number of subjects exceeding MDC (%)		7/10 (70%)^+^		2/10 (20%)

Abbreviation: MDC, minimal detectable change.

*p < 0.05 for intra-group comparison.

^+^p < 0.05 for inter-group comparison.

The results of gait performance are shown in Table 5. Participants demonstrated significant improvement in speed (p = 0.047) and stride length (p = 0.028) after Ai Chi training while participants showed significant improvement in stride length (p = 0.009) after control training. However, there were no significant inter-group differences.

**Table 5:** Gait performance.

Parameters	Ai Chi group (n = 10)			Control group (n = 10)		
	Pre	Post	Change value^a^	Pre	Post	Change value^a^
Speed (cm/s)	61.8 ± 14.6	68.7 ± 16.2*	12.5 ± 18.9%	60.0 ± 27.5	68.1 ± 25.4	20.6 ± 24.1%
Cadence (steps/min)	82.7 ± 14.9	83.9 ± 18.64	0.9 ± 12.7%	88.1 ± 28.3	90.3 ± 25.1	5.6 ± 13.7%
Stride length (cm)	82.1 ± 22.9	89.9 ± 22.4*	11.9 ± 15.4%	77.3 ± 20.8	87.8 ± 18.7*	14.2 ± 10.7%
Stride time (sec)	1.5 ± 0.3	1.5 ± 0.5	0.8 ± 15.6%	1.7 ± 1.1	1.5 ± 0.8	−5.9 ± 11.7%
Stride length variability (%)	6.8 ± 2.7	5.9 ± 1.3	−7.7 ± 22.5%	6.7 ± 3.1	5.4 ± 3.4	−11.8 ± 48.4%
Stride time variability (%)	7.7 ± 4.0	5.8 ± 3.0	−18.9 ± 36.8%	7.6 ± 4.9	6.4 ± 3.6	−5.9 ± 38.5%
Spatial asymmetry ratio	1.1 ± 0.2	1.2 ± 0.2	4.8 ± 28.1%	1.1 ± 0.1	1.0 ± 0.1	−1.4 ± 5.4%
Temporal asymmetry ratio	1.3 ± 0.3	1.3 ± 0.2	3.1 ± 35.6%	1.3 ± 0.4	1.2 ± 0.2	−0.5 ± 16.4%

^a^Change values were calculated by subtracting the pre-training data from the post-training data divided by pre-training data.

*p < 0.05 for intra-group comparison.

Both groups also improved significantly in lower extremity motor control as indicated by Fugl-Meyer assessment (FMA) after training (p = 0.001, p = 0.009, for Ai Chi and control group respectively), and Ai Chi group improved more than the control group (p = 0.030) (Table 6).

**Table 6:** The lower extremity motor control indicated by Fugl-Meyer assessment.

	Ai Chi (n = 10)		Control (n = 10)	
	Pre	Post	Pre	Post
FMA	22.0 ± 3.9	28.7 ± 4.2*	21.7 ± 5.7	24.6 ± 7.7*
Change score		6.7^+^		3.3

*p < 0.05 for intra-group comparison.

^+^p < 0.05 for inter-group comparison.

## Discussion

Our results showed that the Ai Chi exerted superior effects on anteroposterior dynamic balance control and lower extremity motor control than conventional water-based exercise in individual with chronic stroke. The gait speed improved only after Ai Chi training. However, both Ai Chi and conventional water-based exercise improved the lower extremity motor control and BBS. We also noted that no drop-out or adverse event reported during the 6-week study period for both groups.

Ai Chi is one of the special of aquatic exercise resembling Tai Chi on land, however, its effects on balance has not yet been established in people with stroke. In this study, we demonstrated that Ai Chi exerted significant effects on center of gravity (COG) displacement, especially in anteroposterior direction, and such significant effects cannot be achieved by conventional aquatic exercise. Two displacements of COG were recorded during LOS test, EPE and MXE. As EPE is the distance travelled at the first attempt which might indicate the confident excursion of the subjects. MXE, on the other hand, is the maximal result after several attempts. Therefore, the difference between EPE and MXE may imply the strategy and confidence of the subjects during the COG displacement. In current study, improvement of EPE was greater than MXE in anteroposterior direction after Ai Chi training, which may indicate that the confidence for further anteroposterior displacement has been established. According to Eng *et al*., improvement in COG control along the anteroposterior axis was more difficult than in lateral axis for individuals with chronic stroke^30^. In addition, the anteroposterior margin of stability is critical when the post-stroke patients walk or encounter destabilizing environment^31^. Taken together, the increased anteroposterior excursion after Ai Chi training may result in faster walking speed as demonstrated in the present study.

Though BBS could be improved by both water-based programs, only Ai Chi group has reached MDC significantly. Since BBS is a clinical test, reaching MDC suggested “true” change after intervention^32^. Balance impairment is one of the most important internal factor associated with fall^33^, and BBS is a sensitive measure for identifying not only balance deficits but also fall risk^34^. We further analyzed the decrease of fall risk in both groups. The latest cutoff score of increased fall risk in stroke was set at 49 (score ≦ 49)^35^. Changing from BBS ≦ 49 to BBS > 49 indicated the decreased fall risk. At baseline, 8 people in Ai Chi group had higher fall risk and 5 (62%) of them decreased the fall risk. On the other hand, 5 people had higher fall risk in control group, but only 1 (20%) has decreased the fall risk.

Our results of both group were in line with previous studies stating that aquatic therapy can improve lower extremity function and balance performance^27,36^. However, our results further indicated that Ai Chi was more effective than conventional water-based exercise in these two outcome measures. Such superior effect of Ai Chi may be due to that Ai Chi incorporated closed kinetic chain (CKC) exercise while conventional exercise often used open kinetic chain (OKC) movements. CKC exercise involved multiple joints and coordinated muscles while OCK usually isolated one joint. Lower extremity CKC exercise facilitated ankle neuromuscular control superiorly and appeared to be more effective in weight translation^37^. Integrating feedback from the entire lower extremity and further simulating the mechanoreceptors were documented during CKC exercise^38^. With foot sole fixed on the ground, CKC exercise required much eccentric control of knee extensor, as well as calf muscle stretching. Consequently, tibialis anterior could generate higher force and work more effectively^39^. The above mentioned benefits of CKC exercise may explain the superior effects of Ai Chi to conventional water-based exercise in present study.

Another noticeable difference was the movement composition. Conventional water-based exercise was impairment-emphasizing using single-joint movement to focus on exercising the affected limbs, while Ai Chi involved coordinated movements. In particular, the second half movements of Ai Chi (movement #7 to #16) were classified as total coordinated body movements^23^. Four of which (movement #10 to #13) highlighted weight shifting in the anteroposterior axis, and only two, sideways (movement #9, #14). The rest of the movements (movement #8, #9, #15) stressed axial rotation of the trunk and whole body. The last movement (movement #16) required more advanced control of the trunk and lower extremity. Albeit these movements, a diversity of positions were embedded during Ai Chi exercise, including static wide stance, lunge, one-leg stance, and ultimately dynamic crossing steps and jump-landing. Transition of positions was accounted for effective balance training^17^. It was consistent with our results where weight translation has improved significantly in Ai Chi group, particularly in anteroposterior axis, and the result was transferred to the improved BBS.

In this study, we further noted that the directional control (DCL) did not improve as the MXE and EPE did after Ai Chi training. According to motor learning principles, a movement consolidation includes kinematic accuracy, which was acquainted from a large scale of trials and errors, and the later-coming dynamic effects of force^40^. We thus speculated that the increased displacement (indicated by MXE and EPE) but not the kinematic accuracy (indicated by DCL) may reflect the movement consolidation has not yet been established.

The small sample size of our study is one of the limitations. A larger randomized controlled clinical trial is needed to validate the reported benefits of the Ai Chi intervention for people with chronic stroke. Despite the small sample size, the effect size is relatively strong for our outcomes (LOS: 0.57; BBS: 0.44)^41^. In addition, the therapist was not blinded to the exercise group and, although unavoidable, this limitation may introduce bias. Also, the decrease in fall risk was reported according to the BBS in present study, however, the reduction of fall incidence after training warrants further follow-up. Furthermore, it should be noted that both Ai Chi and conventional water-based exercise included gait training to enhance the effects of exercise training. However, this may dilute the distinction between the Ai Chi and conventional water-based exercise.

This is the first study comparing the effects of Ai Chi with conventional water-based exercise in individuals with stroke. With a total of 18 sessions in 6 weeks, Ai Chi resulted in better improvement in anteroposterior balance control and lower extremity motor function than conventional water-based exercise. Both water-based exercise improved BBS score, however, only Ai Chi training decreased the possible fall risks.

## Methods

### Design

This was a single-blinded (assessor blinded) randomized controlled trial with pre- and post-test. The randomization was block randomization with the block of 4 and was conducted via sealed envelope drawn by a person who was not involved in the study. Participants with chronic stroke were recruited and referred by doctors in a general hospital. Stroke diagnosis, age, gender, stroke type, lesion side, and duration of hemiparesis were obtained from patient interviews and medical chart. Subjects who met the selection criteria and consented to participate were randomly allocated to the experimental group or the control group. All participants were assessed before (pre) and after completing the 6 weeks of intervention (post) by a physical therapist who was blinded to the group allocation. Assessment included balance performance, gait performance, and motor control of lower extremity. Participants in experimental group received Ai Chi intervention and control group received conventional water-based exercise for 60 min each session, 3 sessions per week for a total of 6 weeks.

Participants provided written informed consent of study procedures approved by ethical committees at Cheng Hsin General Hospital and National Yang-Ming University. This trial was registered in 10/06/2016 at http://www.anzctr.org.au/ (ACTRN12616000769482) and conformed to the CONSORT checklist. All experiments were performed in accordance with relevant guidelines and regulations.

### Participants

The inclusion criteria of this study were: (1) age between 20 and 80 years old, (2) 6 months post first-ever stroke with unilateral motor deficits, (3) ability to walk independently for at least 15 meters with or without use of walking aids, and (4) a score of >24 on the mini-mental state examination (MMSE). The exclusion criteria were: (1) receiving water-based exercise on a regular basis, (2) unstable vital signs (resting heart rate >100 bpm, body temperature >38 °C, or respiration rate >20 breaths per minute), (3) history of major cardiovascular event (such as myocardial infarction, heart failure, or endocartditis), (4) any hydrotherapy contraindication, such as pregnancy, incontinence, open wound or intubation, and (5) history of other diseases known to possibly interfere with participating the study, such as diabetic neuropathy or uncontrolled hypertension.

### Outcome measures

The primary outcome of this study was balance performance including dynamic balance and functional balance. Dynamic balance was assessed by LOS test using the SMART Balance system (NeuroCom International, Inc, USA). To assess the LOS, the subject stood on the force plate and shifted his/her center of pressure (COP) to reach maximal distance in the target direction as quickly and accurately as possible without moving the feet. Four directions (forward, backward, non-affected side, and affected side) were assessed in random order. MVL, EPE, MXE, and DCL were collected during the LOS test in this study^7,8^. MVL is defined as the average speed in a specific direction. EPE is the distance covered in the very first attempt towards the target, expressed in percentage. MXE is defined as the farthest distance traveled by the COP during the trial. DCL is defined as the amount of movement in the intended direction minus the amount of extraneous movement. A DCL score of 100% indicates that the participant does not deviate from a straight path during the test^42^. The anteroposterior direction and lateral direction were calculated and reported in this study. In anteroposterior direction, displacement percentage of EPE and MXE was the sum of forward and backward direction; MVL and DCL was the average. In lateral direction, displacement percentage of EPE and MXE was the sum of affected and non-affected direction; MVL and DCL was the average. Functional balance was assessed by BBS. BBS is a valid and reliable scale containing 14 items from a sitting position to standing on one foot. Each item is scored from 1 to 4 with a highest possible total score of 56. Higher score stands for better balance performance^32^.

Secondary outcomes included gait performance and motor control of lower extremity. Gait performance was assessed by GAITRite system (CIR system, Inc, USA). Participants were asked to walk 3 times on a GAITRite carpet with sensors attached underneath. The carpet was 3.66 m in length and 0.61 m in width. Spatio-temporal parameters were recorded and calculated. Gait parameters of interest in this study included speed, cadence, stride length, stride time, stride length variability, stride time variability, spatial asymmetry ratio, and temporal asymmetry ratio^43^. Variability was expressed by standard deviation/mean ×100% to indicate the consistency of gait pattern^44^. Asymmetry ratio was quantified by the percentage as follows: paretic side/non-paretic side ×100%, with step length indicating for spatial parameters and step time for temporal^45^. The averaged value of 3 trials were used for data analysis. The lower extremity motor control was evaluated by FMA scale which has been reported with good reliability for stroke patients^46,47^. The total score of lower extremity part is 34, and higher score indicates better control of lower extremity. All the data were collected in Neurological Physiotherapy Lab in National Yang-Ming University.

### Intervention programs

Participants in experimental group and control group received exercise program in a 120 cm deep, 35 °C therapeutic pool with therapist and participant ratio 1:2. Both groups were instructed by an experienced aquatic physiotherapist with Ai Chi certificate.

The participants in the experimental group received 16-kata Ai Chi for warm-up and main exercise. Each kata and its movement characteristics were described in Supplementary Table S1. In each session, participants practiced the first 3 katas of Ai Chi as warm-up for 15 min. Those katas were mainly for breathing control and symmetrical arm movements in wide stance position. After the warm-up exercise, participants performed 3–4 katas with 10–15 repetition for each katas as the main exercise program for 30 min. Gait training for another 15 min was administered to conclude the intervention. The difficulties of the katas progressed every week until week 5. In the last week, participants were asked to practice a full Ai Chi program as the main exercise program^23^. The progression was shown in Supplementary Table S2.

Participants in the control group also started a session with 15 min warm-up by practicing active movement of the affected side. The 30-min main exercise included stretching exercise and resistance exercise for affected leg. For the stretching exercise, the participants were in a sitting position in water with 90-degree hip and knee flexion and feet on the floor for 30 seconds and then stood up for 5 times. The participants were allowed to lean back on the wall or to use the handrail for balance. Resistance training included four combinations of movements: hip flexion-extension, hip abduction-adduction, knee flexion-extension, ankle dorsiflexion-plantar flexion. In hip and knee movements, a flotation aid was placed and fixed around the ankle of the affected side. Ankle movement was floatation-free. Participants were asked to exercise the affected leg as fast as they can without losing standing balance. The therapist provided verbal feedback and manual guidance as needed during movement. Each movement was practiced 10 times as one set, and the participants needed to practice 3 sets for the resistance training. During the first set of practice, the participants were asked to hold the handrail with the non-affected hand. During the second and the third set of practice, the participants were encouraged to practice without holding the handrail. Gait training for 15 min was administered to conclude the intervention.

### Statistical analysis

A sample of 14 participants was calculated to be sufficient for power of 0.80 and a two-tailed alpha level of 0.05 to detect a between-group difference in BBS (effect size 1.03)^27^. To account for some attrition, 20 participants were recruited.

All the data was analyzed by SPSS 24.0. Descriptive statistics were generated for all variables, and distributions of variables were expressed as mean ± standard deviation or frequency. Normal distribution of outcomes cannot be confirmed by Shapiro-Wilk test, and thus inter-group difference of baseline data was analyzed by Mann-Whitney U test for continuous variables or *X*^2^ test for nominal scales. Mann-Whitney U test was used to compare the percentage of changes of outcomes between groups, and Wilcoxon signed rank test for within group comparisons. Statistical significant level was set at 0.05.

## Supplementary information

Supplementary information.

## Data Availability

The datasets generated during and/or analyzed during the current study are available from the corresponding author on reasonable request.
