# Supplementary material for: The effects of Ai Chi for balance in individuals with chronic stroke: a randomized controlled trial
Source: Sci Rep. 2020 Jan 27;10:1201. doi: 10.1038/s41598-020-58098-0 (PMC6985180; doi:10.1038/s41598-020-58098-0)
Supplement: Supplementary file 1 — Supplementary information. [file 41598_2020_58098_MOESM1_ESM.pdf]

The effects of Ai Chi for balance in individuals with chronic stroke: a randomized controlled trial

Pei-Hsin Ku[1], PT, Szu-Fu Chen[2], PhD, MD, Yea-Ru Yang[1], PhD, PT, Ta-Chang Lai [3*], MD, Ray-Yau Wang[1*], PhD, PT

[1]Department of Physical Therapy and Assistive Technology, National Yang-Ming University, Taipei, Taiwan, ROC

[2]Department of Physical Medicine and Rehabilitation, Cheng Hsin General Hospital, Taipei, Taiwan, ROC

[3]Department of Neurology, Cheng Hsin General Hospital, Taipei, Taiwan, ROC

* Corresponding authors:

Ray-Yau Wang, PhD, PT

Professor,

Department of Physical Therapy and Assistive Technology,

National Yang-Ming University, No. 155, Sec.2, Li-Nong Street,

Taipei, zip code: 112, Taiwan, ROC

Tel: +886-2-2826-7210

Fax: +886-2-2820-1841

Email: rywang@ym.edu.tw

Ta-Chang Lai, MD

Doctor,

Department of Neurology,

Cheng Hsin General Hospital, No.45, Cheng Hsin Street,

Taipei, zip code: 112, Taiwan, ROC

Tel: +886-2-2826-4400

Email: ch6506@chgh.org.tw

Supplementary Table S1. Movement description of 16-kata Ai Chi

#	Kata (Form)	Movement aim	Position	Movement action
1	*Contemplating*	Breathing control	Wide stance, weight evenly distributed	Both forearms alternatively supinated and pronated at 90-degree shoulder flexion.
2	*Floating*			Both arms flexed from 0 to 90 degrees and back down.
3	*Uplifting*			Both arms abducted from 0 to 90 degrees and back down.
4	*Enclosing*	Trunk stability		Both arms horizontally adducted until hands touching together and open again.
5	*Folding*			Both elbows maintained 90-degree flexion at side, alternating with shoulder external and internal rotation.
6	*Soothing*			Both arms abducted at 90 degrees, moving one arm horizontally to the other side and reverse.
7	*Gathering*	Basic coordination	Lounge position	Both arms flexed at 90 degrees, horizontally abducting the arm contralateral to the forward leg.
8	*Freeing*		Change between wide stance and lounge position	"Freeing" consisted of the movements in "soothing" followed by movements in "gathering".
9	*Shifting*		Wide stance, weight reciprocally shifted to left and right	Arm movements were the same as "soothing" but with weight shifting laterally.
10	*Accepting*		Lounge position, weight constantly shifted forward and backward	Both arms horizontally abducted and adducted combined with weight shifting.
11	*Accepting with grace*		One leg standing	Movements are the same as "accepting", but the forward leg should be off the ground during weight shifting backward.

12	*Rounding*			Movements are similar to "accepting". But when the weight was shifted forward, back leg was simultaneously lifted up and flexed forward.
13	*Balancing*			Both arms flexed forward while one leg extended backward, and reverse.
14	*Flowing*	Advanced coordination	Walking with cross steps	Arms alternatively crossed before abdomen and open during cross steps.
15	*Reflecting*		Walking with cross steps and rotation	Arm movements are the same as "flowing", but the steps integrated a 180-degree-turn after the crossed step.
16	*Suspending*		Jumping	Subjects crossed the leg with a gentle jump, and uncross with a 180-degree-turn

Supplementary Table S2. The six-week intervention program for Ai Chi group

	Movement aim	Week 1	Week 2	Week 3	Week 4	Week 5	Week 6
Warm up	Breathing control	#1, #2, #3					
Main exercise	Trunk stability	#4, #5, #6	#4, #5, #6	#5, #6	#5, #6		Practice #1-16 in sequence
	Basic coordination		#7	#8, #9	#10, #11	#12, #13	
	Advanced coordination					#14, #15, #16	
Gait training	Forward and backward walking, sideways, marching, high-knees, walking in normal speed and walking fast						

The # indicates the Kata (Form) described in Supplementary Table S1.
